# Involvement of CBF in the fine-tuning of litchi flowering time and cold and drought stresses

**DOI:** 10.3389/fpls.2023.1167458

**Published:** 2023-06-12

**Authors:** Xiaozhen Shan, Yun Yang, Shuoqi Wei, Chao Wang, Weishi Shen, Hou-bin Chen, Ji-yuan Shen

**Affiliations:** Key Laboratory of Biology and Germplasm Enhancement of Horticultural Crops in South China, Ministry of Agriculture/Guangdong Litchi Engineering Research Center, South China Agricultural University, Guangzhou, China

**Keywords:** litchi, flower, cold, CBF, MFT

## Abstract

Litchi (*Litchi chinensis*) is an economically important fruit tree in southern China and is widely cultivated in subtropical regions. However, irregular flowering attributed to inadequate floral induction leads to a seriously fluctuating bearing. Litchi floral initiation is largely determined by cold temperatures, whereas the underlying molecular mechanisms have yet to be identified. In this study, we identified four *CRT/DRE BINDING FACTORS* (*CBF*) homologs in litchi, of which *LcCBF1*, *LcCBF2* and *LcCBF3* showed a decrease in response to the floral inductive cold. A similar expression pattern was observed for the *MOTHER OF FT AND TFL1* homolog (*LcMFT*) in litchi. Furthermore, both LcCBF2 and LcCBF3 were found to bind to the promoter of *LcMFT* to activate its expression, as indicated by the analysis of yeast-one-hybrid (Y1H), electrophoretic mobility shift assays (EMSA), and dual luciferase complementation assays. Ectopic overexpression of *LcCBF2* and *LcCBF3* in *Arabidopsis* caused delayed flowering and increased freezing and drought tolerance, whereas overexpression of *LcMFT* in *Arabidopsis* had no significant effect on flowering time. Taken together, we identified LcCBF2 and LcCBF3 as upstream activators of *LcMFT* and proposed the contribution of the cold-responsive CBF to the fine-tuning of flowering time.

## Introduction

Litchi (*Litchi chinensis* Sonn.) is a subtropical evergreen fruit tree native in China (Hu et al., 2022), and is also widely cultivated in subtropical regions due to its high nutritional values, attractive appearance, and striking tastes. Litchi plants mostly flower and bear in the terminal shoots, whereas its irregular flowering attributed to the inadequate floral induction results in serious fluctuating production (Menzel, 1983). Perception of a period of low temperature is largely required for litchi floral initiation, and temperatures above 20°C are highly detrimental to the floral induction (Menzel and Simpson, 1988). Although the floral initiation of litchi can be affected by drought that appropriate drought prior to cold induction can reduce the requirement of low temperatures for litchi floral induction, but litchi plants cannot complete floral induction in the absence of prolonged exposure to low temperatures (Stern and Gazit, 2010; Shen et al., 2016).

Flowering is an important transition from the vegetative to reproductive phase during the plant’s life cycle in response to both environmental and endogenous signals, such as temperature, photoperiod, and age (Amasino, 2010; Song et al., 2013; Wang, 2014). Based on the genetic and physiological analyses in *Arabidopsis thaliana*, floral induction is regulated by four major genetic pathways, including day-length, vernalization, autonomous, and gibberellin-dependent pathways. These pathways fine-tune a set of genes to switch on/off the biosynthesis of florigen encoded by *FLOWERING LOCUS T* (*FT*), eventually determining the exact flowering time (Amasino, 2010; Song et al., 2013; Wang, 2014).

*FT* is a member of the phosphatidylethanolamine-binding protein (PEBP) family that can be clustered into three subfamilies, including *FT*-like, *TERMINAL FLOWER 1* (*TFL1*)-like, and *MOTHER OF FT AND TFL1* (*MFT*)-like clades. The *MFT*-like clade is proposed to be an ancestor of the family (Hedman et al., 2009). In general, PEBP members mostly interact with FLOWERIING LOCUS D (FD) protein, instead of directly bind to DNA, to manipulate the expression of downstream targets (Abe et al., 2005; Hanano and Goto, 2011; Wickland and Hanzawa, 2015). TFL1 and its homologs are best known for their opposite roles to FT in regulating flowering time, and act as key players in the formation of inflorescence architecture (Hanano and Goto, 2011; Wickland and Hanzawa, 2015). In contrast, *MFT* is strongly expressed in seeds and promotes seed dormancy (Xi et al., 2010; Nakamura et al., 2011), while the role of *MFT* in the determination of flowering time is still ambiguous. For instance, overexpression of *MFT* in *Arabidopsis* resulted in slightly early flowering, but the loss of *AtMFT* function barely led to an obvious phenotype in flowering, proposing a redundant role for *MFT* in *Arabidopsis* flowering (Yoo et al., 2004). In kiwifruit, two *MFT*-like genes show no influence on flowering time (Voogd et al., 2017). Interestingly, ectopic overexpression of *AcMFT*, a *MFT* homolog from a non-flowering plant *Adiantum capillus-veneris*, promotes the floral initiation and partially rescues the late-flowering defect in *ft-1* mutants in *Arabidopsis* (Hou and Yang, 2016). However, *OsMFT1* clearly acts as a suppressor of flowering time but a promoter of panicle architecture in rice (Song et al., 2018). Similar studies demonstrated that ectopic overexpression of *HbMFT1* (Bi et al., 2016) and *DnMFT* (Li et al., 2012) in *Arabidopsis* causes delayed flowering. Despite the importance of PEBP members in plant development, their upstream regulators remain largely unknown.

Cold activates a range of so-called *cold-regulated* (*COR*) genes encoding proteins that enhance the freezing tolerance of plants. Such *COR* genes share a cis-acting DNA regulatory element in their promoters, namely, the C-repeat/dehydration responsive elements (CRT/DRE). CRT/DRE BINDING FACTORS (CBFs) serve as the key upstream regulators of the cold response pathway in plants (Stockinger et al., 1997). CBF/DREB1 is a class of AP2/ERF (APETALA2) transcription factor proteins that are extensively involved in responses of plants to external stresses such as cold and drought (Novillo et al., 2004; Thomashow, 2010; Zhou et al., 2011; Nakashima et al., 2014). There are four *CBF* homologs identified in *Arabidopsis*, of which *CBF1*, *2*, and *3* can be significantly induced by cold, and improve freezing tolerance (Stockinger et al., 1997; Gilmour et al., 2004). In contrast to the three *CBF* homologs, *AtCBF4* is up-regulated by drought instead of by low temperatures (Haake et al., 2002). It is noteworthy that *AtCBF1* and *AtCBF3* are modulated distinctly than *AtCBF2*, and AtCBF2 can negatively regulates the expression of *AtCBF1* and *AtCBF3* (Novillo et al., 2004; Novillo et al., 2007).

CBFs not only play an important role in plant response to abiotic stresses, but also participate in plant flowering regulation. In *Arabidopsis*, constitutively expression of *AtCBF1* or *AtCBF4* leads to late-flowering (Haake et al., 2002; Achard et al., 2008). The condition of short or intermittent low temperatures delays the flowering of *Arabidopsis thaliana* through the activation of the expression of *FLC* (*FLOWERING LOCUS C*) induced by CBF*s*, and overexpression of *CBF1/2/3* in *Arabidopsis thaliana* can significantly induce the expression of *FLC* (Gilmour et al., 2004; Seo et al., 2009). Furthermore, *AtFLC* was proved to be directly activated by INDUCER OF CBF EXPRESSION 1 (ICE1), an upstream activator of *CBF*s, revealed by the analysis of *ice1* mutants in which *AtFLC* was significantly down-regulated, and the time to flowering was significantly promoted (Lee et al., 2015). However, the CBF-dependent regulatory pathways in plant flowering are not fully understood.

In this research, the *CBF* homologs were isolated and identified in the litchi genome. The expression profiles of *CBF* genes in responses to low temperature were investigated. Changes in the expression of flowering genes were also examined during the floral induction. We showed the transcriptional regulation of *LcMFT* by LcCBFs. Taken together, we propose a new strategy for litchi floral induction in a CBF-dependent manner.

## Materials and methods

### Plant materials and growth conditions

Potted air-layered litchi trees (*Litchi chinenesis* cv. Feizixiao) grown in the greenhouse at South China Agricultural University in Guangzhou were used in this study. The terminal shoots of these plants were pruned and turned to be fully mature prior to the cold treatment. The plants were treated at 15°C with the approximately 25% soil moisture for 40 days under nature lights. After the floral induction treatment, the temperature increased to 25°C for flower development. Leaves in the terminal shoots were collected 0, 10, 30 and 40 days during the cold treatment, and the leaf tissues were frozen in the liquid nitrogen as soon as possible, subsequently stored at -75°C for further studies.

### Overexpression of litchi genes in *Arabidopsis*

To obtain overexpression constructs, the coding sequence of *LcCBFs* and *LcMFT* was linked to the expression vector *pCAMBIA*-1302 using the homologous recombination method. The recombinant plasmid was introduced into the *Agrobacterium tumefaciens* strain GV3100. Subsequently, the positive bacteria were transformed into *Arabidopsis thaliana* ecotype Columbia (Col) using the pollen tube introduction method to obtain transgenic *Arabidopsis* plants. Seeds of the transformed plants were germinated and selected on Murashige and Skoog (MS) agar media in the presence of Hygromycin (Hyg). One-week-old seedlings were transferred into soil (nutrient soil: vermiculite: perlite=4:1:1), and plants were grown in growth chambers at 23°C under long daylength condition (light/dark for 16h/8h). Homozygous transgenic plants were used in the following experiments.

### RNA isolation and quantification

The total RNA of the collected plant materials was extracted using the RNA Prep Pure Polysaccharide Polyphenol Plant TOTAL RNA Extraction kit (Beijing Tiangen Biochemical Technology). The first strand of cDNA was reverse-transcribed with the HiScript^®^ II qRT SuperMix kit. cDNA samples were used as templates for the following real-time quantitative PCR (Q-PCR) assays. Triplicate quantitative assays and three biological replicates were performed on Bio-Rad iQ5 Optical System using the SYBR Green PCR Master Mix (Bio-Rad). The specificity of each pair of primers was evaluated before Q-PCR analysis and primers were listed in the **Supplementary Table 1**. The relative gene expression was calculated using the 2^-△△CT^ method (Livak and Schmittgen, 2001), and values are indicated as mean ± standard error.

### Plant freezing tolerance test

The freezing tolerance test of transgenic *Arabidopsis* plants over-expressing *LcCBF*s was conducted as previously documented (Li et al., 2018). 4-week-old seedlings grown in the soil were adopted to cold acclimation at 4°C for 3 d, and then were transferred into a freezing container set at 0°C with a decrease of 1°C/h to -8°C for 3 h. After the freezing treatment, the plants were put at 4°C in the dark for 12 h to recover and then were transferred to 22°C. The survival rate was scored three days later. Two transgenic lines for each gene were used in the experiment and at least three biological replicates per line were examined.

### Plant drought tolerance test

The drought tolerance test of transgenic *Arabidopsis* plants over-expressing *LcCBF*s is performed according to Siddiqua’s method with slight modifications (Siddiqua and Nassuth, 2011). 30-day-old *Arabidopsis* plants from wild-type (WT) and transgenic groups were used in this study. The soil substrates in the pots were saturated with water, and 12 h later plants were subjected to a 16 day-drought treatment without any watering. During the drought treatment, the environmental conditions were maintained at 23°C with the relative humidity is approximately 50%. After that, drought-treated plants were re-watered, and the survival ratio and phenotypes were analyzed after 7 days. By contrast, the control plants were watered for the whole duration of the experiment. Two transgenic lines for each gene in the experiment and at least three biological replicates per line were examined.

### Whole-Genome identification of CBF and PEBP homologs

The CBF and PEBP protein sequences of *Arabidopsis* and litchi were obtained from TAIR (http://www.arabidopsis.org/) and SapBase (http://www.sapindaceae.com/) databases, respectively. The well-known CBF and PEBP sequences in *Arabidopsis* were used as queries to search candidate homologs by BLAST against the litchi genome with TBtools software (v1.09854; https://github.com/CJ-Chen/TBtools/releases). In addition, the CBF and PEBP Strubbelig-Receptor Family (SRF) domain (PF00847 and PF00319, respectively) were used to identify the genes using a Hidden Markov Model Search (Guan et al., 2021). All the predicted sequences were further confirmed using Conserved Domain Database (CDD) (http://www.ncbi.nlm.nih.gov/cdd/) and Simple Modular Architecture Research Tool (SMART, http://smart.embl-heidelberg.de/) to search for conserved domains. Finally, CBF and PEBP candidate genes were manually examined to remove redundant and incomplete sequences.

### Measurement of *Fv/Fm* and electrolyte leakage (EL)

*Fv/Fm* (ratio of variable to maximum fluorescence, *Fm*, fluorescence maximum) value, reflecting the quantum efficiency of photosystem II, is widely used to illustrate the effects of environmental stresses on the plant. In this study, *Fv/Fm* values of leaves from plants subjected to cold or drought treatments were examined using a chlorophyll fluorescence imaging system (Imaging-PAM, Walz, Germany). Prior to the measurements, the whole plant was adapted to darkness for 20 min. Three individual plants of each line were used for *Fv/Fm* measurements.

For measuring of electrolyte leakage of leaves, two fresh leaves (approximately 0.2 g) of *Arabidopsis* plants from each group were cut and put into a 15 mL centrifuge tube with 5 mL double distilled H_2_O. The tubes were shaken at 150 rpm and 25°C for 2 h, and then the electrolyte leakage of the solution (L1) was measured using a conductivity meter (CON2700, Eutech instrument). After that, the solutions were boiled for 20 min, and the electrolyte leakage of which (L2) was measured again after they were cooled down to room temperature under running water. The relative electrolyte leakage was calculated as follows: EL (%) = (L1/L2) × 100%.

### Dual luciferase complementation assay

The luciferase assay was conducted *in vitro* according to the method as described (Shan et al., 2014). The coding sequence (CDS) of *LcCBF*s and the promoter of *LcMFT* were amplified with the primers listed in **Supplementary Table 1**. The CDS of *LcCBF*s were cloned into the pGreen II 0029 62-SK vector, while the promoter of *LcMFT* was recombined into the pGreen II 0800-LUC vector. The two sets of recombinant plasmids were electroporated into *Agrobacterium tumefaciens* (GV3101). Thereafter, positive bacterial cultures were selected and prepared with the infection buffer (200 mM acetosyringone, 10 mM MES, 10 mM MgCl2, pH 5.6) to an OD_600_ of 0.8 at 28°C. Mixtures of transformed *A. tumefaciens* expressing the promoter of *LcMFT* and the full CDS of *LcCBF*s were co-infiltrated into fully expanded leaves of approximately 3-week-old tobacco (*Nicotiana benthamiana*) plants with a needless syringe, while the mixtures of empty pGreen II 0029 62-SK and *LcCBF*-Luc vectors were co-infiltrated into the other half leaves as negative controls. The transformed tobacco plants were grown at 23°C in a growth chamber (16 h daylight). Three days later, the infected tobacco leaves were collected for measurements of renilla luciferase and firefly luciferase activities using a dual luciferase assay system (Promega, Madison, WI, USA), and the measurements were conducted on a GLO-MAX 20/20 luminometer (Promega, Madison, WI, USA). For each DNA-protein binding assay, two independent experiments and at least three biological replicates per experiment were performed.

### Yeast one-hybrid (Y1H) assay

Yeast-one-hybrid (Y1H) assays were conducted as previously described by Xiao et al. (2019) to detect the potential DNA-protein interaction, using the Matchmaker™ Gold Yeast One-Hybrid System kit (Clontech, CA, USA). The domain of the *LcMFT* promoter (1119 bp), including one CBF recognition core element (CCGAC), was amplified and inserted into the pAbAi plasmid. Thereafter, the recombinant plasmid was transformed into the competent Y1H Gold strain. The positive yeasts were selected and co-transformed with pGADT7-AD inserted with the coding sequence of the *LcCBF* genes. The binding of transcription factor and promoter was determined based on the growth ability of the yeast cells harboring these co-transformants on SD/-Leu medium in the presence of aureobasidin A (AbA).

### Electrophoretic mobility shift assay (EMSA)

Electrophoretic mobility shift assays were performed as described by Shan et al. (2014). The coding sequence of *LcCBF*s was amplified and fused into pGEX-4T-1 to generate the LcCBF-GST fusion construct. The fusion construct was transformed into *E. coli* strain BL21 cells. After growing to saturation, the transformed cells were collected and treated with 0.2 mM isopropyl β-D-1-thiogalactopyranoside (IPTG), and incubated at 37°C for 3 h to express the fusion protein. The LcCBF-GST fusion protein was purified and collected using a Pierce GST spin purification kit (Thermo Scientific) according to the instructions. The promoter fragments of *LcMFT* used as the probes were synthesized and biotin-labeled using a LightShift chemiluminescent EMSA kit (Thermo Scientific). LcCBF-GST protein and biotin-labeled probes were incubated at room temperatures, while unlabeled cold probes, mutated probes and the GST protein alone acted as competitors and negative controls, respectively. All of the biotin-labeled binding DNA was analyzed by 6% native polyacrylamide gel electrophoresis, and was detected using the chemiluminescence method on a ChemiDoc MP imaging system (Bio-Rad). The primers used in the EMSA assays are listed in **Supplementary Table 1**.

### Statistical analysis

T-test was used for two-sample statistical comparisons in this study. For multiple comparisons, one way analysis of variance (ANOVA) was performed using SPSS software (v.22, IBM) followed by a Duncan test. The significant statistical differences are indicated in each figure.

## Results

### Genome-wide identification and phylogenetic analysis of *CBF* and *MFT homologs*

**Figure 1:**
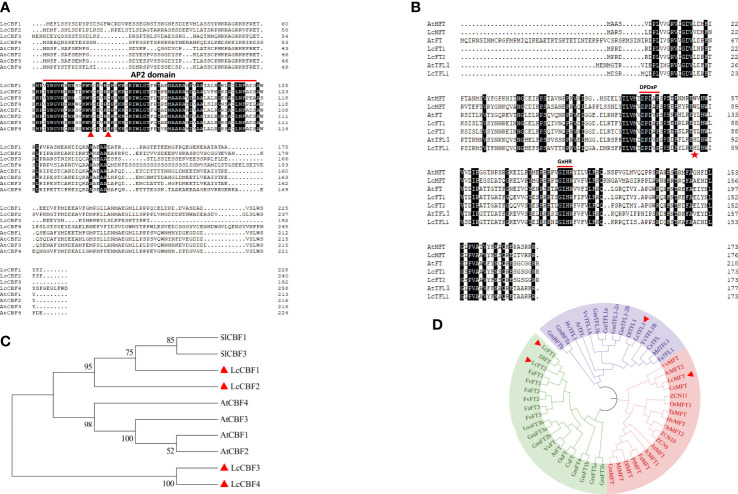
Alignment and sequence analysis of protein sequences encoded by *LcCBF*s and *LcMFT*. **(A)** Alignment of CBF protein sequences from litchi and *Arabidopsis*, and the conserved AP2 domain was indicated with a red line. **(B)** Phylogenetic analysis of CBF protein sequences from litchi (indicated with red triangles), *Arabidopsis thaliana* and tomato. Accession numbers of these protein sequences are shown in **Supplementary Table 2**. **(C)** Alignment of putative PEBP protein sequences from litchi and their homologs. The highly conserved motifs of the PEBP family including D-P-D-x-P and G-x-H-R were indicated with red lines. The conserved position of each subfamily was indicated with a red pentagram. **(D)** Phylogenetic analysis of PEBP protein sequences from litchi (indicated with red triangles) and other species. Accession number and species name of these protein sequences are shown in **Supplementary Table 2**.

Since CBFs play important roles both in freezing tolerance and thermos sensory-pathway regulated flowering, *CBF* genes were identified and investigated in this study. Four litchi *CBF* homologs were identified on the basis of BLAST searches against the litchi genome using *Arabidopsis CBF* genes. The length of proteins encoded by *LcCBF*s arranged from 192 to 259 amino acid residues (**Figure 1A**). According to the multiple alignment of protein sequences from litchi and *Arabidopsis*, all litchi CBF proteins contain a unique AP2 DNA-binding domain of about 60 amino acid residues, which is evolutionally conserved in plants (**Figure 1A**). Furthermore, as shown by the phylogenetic analysis of protein sequences, litchi CBFs are distinguished from the *Arabidopsis* CBF group, whereas LcCBF1 and 2 were classified into a subgroup with tomato CBF homologs that has showed a role in enhancing freezing tolerance (**Figure 1B**) (Zhang et al., 2004; Zhang et al., 2020). Taken together, these results show that the identified litchi CBF sequences are *Arabidopsis* orthologs, but have species-related features.

The PEBP family can be mainly divided into three subclades, including FT, MFT, and TFL1 that primarily act as floral pathway integrators (Hanano and Goto, 2011; Wickland and Hanzawa, 2015). Previously, two members of the FT subgroup were identified and studied in litchi (Ding et al., 2015; Xiao et al., 2019). Here, homologs in the TFL1 and MFT subgroups were identified via protein homology searches of the litchi genome using the full-length protein sequences of PEBP members in *Arabidopsis* and the genome annotation, and found one member for each subclade. Overall comparisons of PEBP protein sequences between litchi and *Arabidopsis* showed that highly conserved motifs including D-P-D-x-P and G-x-H-R, were present in all litchi putative homologs (**Figure 1C**) (Banfield and Brady, 2000). Consistent with the previous study, histidine 88 of TFL1, tyrosine 86 of FT, and tryptophan of MFT exclusively exist at the corresponding positions (indicated with a red pentagram) in each subclade (**Figure 1C**) (Hanzawa et al., 2005). In addition, phylogenetic analysis showed that PEBP family could be clearly divided into three groups, in which litchi MFT and TFL1 exhibited closer genetic relationship to homologs from citrus species than others (**Figure 1D**).

### The expression patterns of *LcCBFs* and flowering-related genes

**Figure 2:**
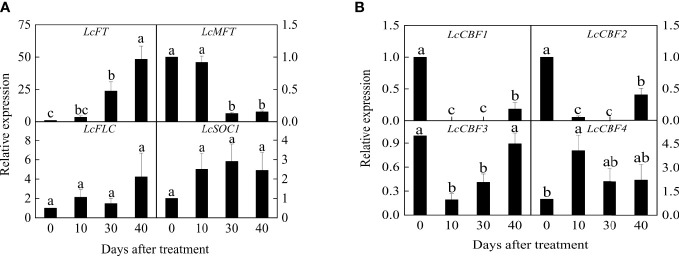
Expression profiles of flowering genes **(A)** and *LcCBF*s **(B)** during the floral induction. Expression values of each gene are calculated as a ratio relative to the beginning of floral induction (0 day), which was set at 1. Values with different letters for gene expression are significantly different at *p* < 0.05. Error bars indicate standard error from six replicates at each time point.

After an exposure to 40 days of low temperature floral induction, all litchi plants were competent to flower. Therefore, the expression of critical flowering-related genes was investigated. As shown by Q-PCR results, the expression of *LcFT1* gene significantly increased during the cold-dependent floral induction, and had approximately fifty times increase at the complete of induction (**Figure 2A**). By contrast, the abundance of *LcMFT* transcript decreased in response to the cold and showed a significant difference 30 days after the treatment. Interestingly, both the expression of *LcFLC* and *LcSOC1* (*SUPPRESSOR OF OVEREXPRESSION OF CONSTANS1*) showed a slight incline during the floral induction, but the changes were not statistically significant (**Figure 2A**).

In addition, the key cold-responsive genes *CBF* homologs were examined in litchi leaves. *LcCBF1* and *LcCBF2* showed a rapid and significant decrease during the floral induction (**Figure 2B**). Similarly, *LcCBF3* decreased in response to the early cold treatment, whereas its expression level was restored at the end of the induction. *LcCBF4* had a significant increase during the ten days, and then decreased, eventually exhibiting no significant changes (**Figure 2B**).

### Regulation of *LcMFT* by LcCBF2 and LcCBF3

**Figure 3:**
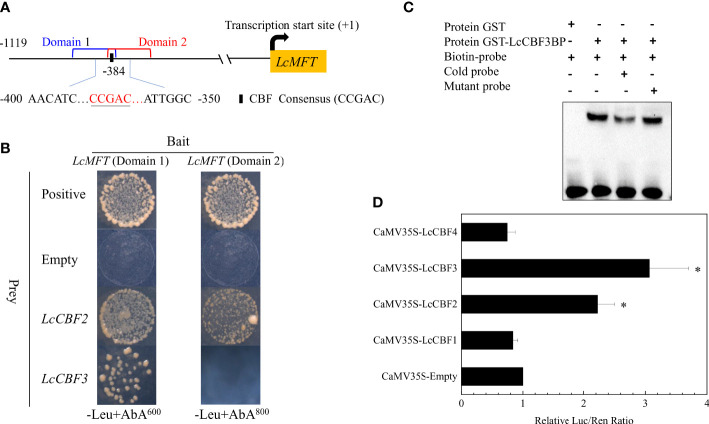
Analysis of interaction of LcCBF proteins and the promoter of *LcMFT*. **(A)** the left panel demonstrates a schematic representation of CBF recognition site (CBFRS) in the promoter of *LcMFT*. **(B)** yeast-one-hybrid (Y1H) assay for direct binding of LcCBF2/3 proteins to the promoter of *LcMFT*. The fragment of the promoter of LcMFT was integrated into the reporter gene AUR1-C (an Aureobasidin A (AbA) resistance gene in yeast). The Y1H reporter strains were transformed with fused plasmids constitutively expressing *LcCBF2* and *LcCBF3* effector, empty (negative control) and *P53* effector (positive control). Interactions between LcCBFs and the promoter of *LcMFT* were determined based on the growth ability of the transformed yeasts on the selective SD medium without Leu in the presence of AbA. **(C)** Electrophoretic mobility shift assays (EMSA) showing the GST-LcCBF3 fusion protein binds to DNA probe from the *LcMFT* promoter *in vitro*. Biotin-labeled probes containing CBFRS and mutated CBFRS, as well as the cold probes (biotin-labeled) were incubated with GST-LcCBF3 proteins, and the DNA-protein complexes were separated on 6% polyacrylamide gels. The probe containing the mutated CBFRS was used to test binding specificity. **(D)** LcCBF2 and LcCBF3 activate the activity of *LcMFT* promoter in the dual Luciferase complementation assay. Transient co-expression of LcCBFs and the promoter of *LcMFT* were performed in tobacco (*N. benthamiana*) leaves as the reporter and effector vectors, respectively. Data show the ratio of firefly luciferase (LUC) and renilla luciferase (REN) activities, and the ratios of LUC to REN of the empty vector (SK) plus the promoter was used as the control and set as 1. The significant difference was determined by Student’s t test and indicated with asterisks at *p* < 0.05.

Despite the few upstream regulators of PEBP family members and the similar expression patterns of *LcCBF*s and *LcMFT*, the regulation of *LcMFT* by LcCBFs was investigated. Yeast one-hybrid (Y1H) analysis was first performed to this aim, due to that there was one CBF recognition element (CCGAC) in the promoter of *LcMFT*. However, there was strong basal activity in yeast cells, when a whole region of the promoter (1119 base nucleotides prior to the transcription start site) was cloned in front of the reporter gene *AUR1-C*. Hence, the promoter of *LcMFT* was truncated into two domains covering the recognition element (**Figure 3A**). After the Y1H reporter strains constitutively expressing the *LcCBF2* or *LcCBF3* effector, the growth of yeast cells including the *LcMFT* promoters was observed in the presence of aureobasidin A to examine the DNA-protein binding. The yeast cells harboring the *LcMFT* promoter could grow well when co-transformed with *LcCBF2* or *LcCBF3* effectors (**Figure 3B**). As evidenced by the Y1H analysis, *LcCBF2* or *LcCBF3* can directly bind to the promoter of *LcMFT*.

Furthermore, electrophoretic mobility shift assay (EMSA) assays were performed using purified recombinant proteins of LcCBFs to confirm the DNA-protein interaction. While no mobility shift was detected in the presence of GST alone, LcCBF3 proteins could bind to the labeled probes including CBF recognition sites derived from the promoter of *LcMFT*, leading to mobility shifts (**Figure 3C**). Additionally, the shifted bands were effectively competed by the addition of unlabeled probes (cold probes), but not by the mutated probes (**Figure 3C**). Unfortunately, we failed in the purification of the LcCBF2-GST protein to conduct the similar EMSA assays.

To verify the DNA-protein binding between LcCBF2/3 and the promoter of *LcMFT in vivo*, we performed a dual-luciferase transient co-transformational assay in tobacco leaves. The interaction between LcCBF2/3 and the promoter of *LcMFT* caused significant increases in Luc activities (**Figure 3D**), indicating that LcCBF2 and LcCBF3 could interact with the promoter of *LcMFT* to activate its transcription.

### Effects of *LcCBF2* and *LcCBF3* on freezing and drought tolerance

**Figure 4:**
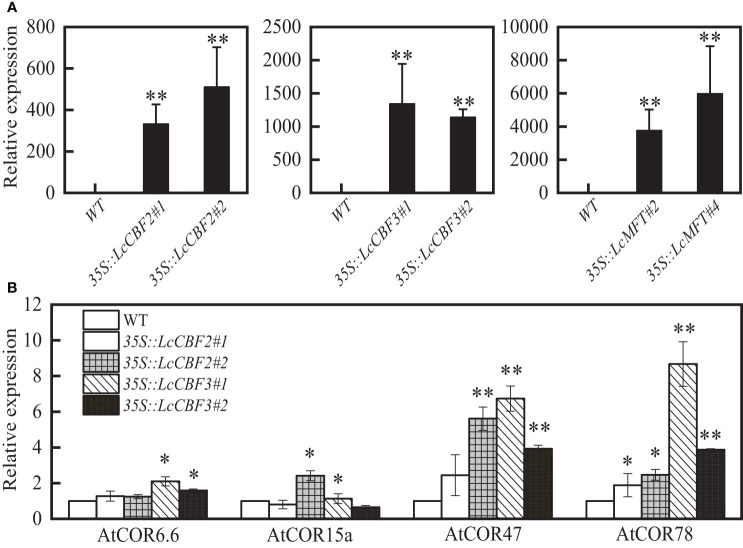
Ectopic expression of *LcCBF2*, *LcCBF3* and *LcMFT* in the wild-type *Arabidopsis thaliana* (Col-0). Identification of the expression level of these litchi genes **(A)** and abiotic tolerance-related *Arabidopsis* genes **(B)** in transgenic plants were determined by Q-PCR. Asterisks indicate significant difference between wild-type and transgenic *Arabidopsis* plants at *P* < 0.05 (*) or *P* < 0.01 (**).

To confirm the putative functions of LcCBF2, LcCBF3, and LcMFT, the constructs containing *LcCBF2/3* or *LcMFT* was driven by the constitutive cauliflower mosaic virus 35S promoter (CaMV 35S), and was then transformed into wild-type *Arabidopsis* (Col-0) to obtain overexpression transgenic plants. Two transgenic T3 lines per gene were selected based on the expression level for further analysis (**Figure 4A**). Furthermore, downstream cold and drought tolerance-related genes in the CBF-dependent regulatory pathway, including *AtCOR6.6*, *AtCOR15a*, *AtCOR47* and *AtCOR78* were examined in transgenic *Arabidopsis* plants (Kasuga et al., 1999; Siddiqua and Nassuth, 2011). As expected, over-expression of *LcCBF2/3* significantly induced the expression level of all four or most of these *COR* genes (**Figure 4B**).

**Figure 5:**
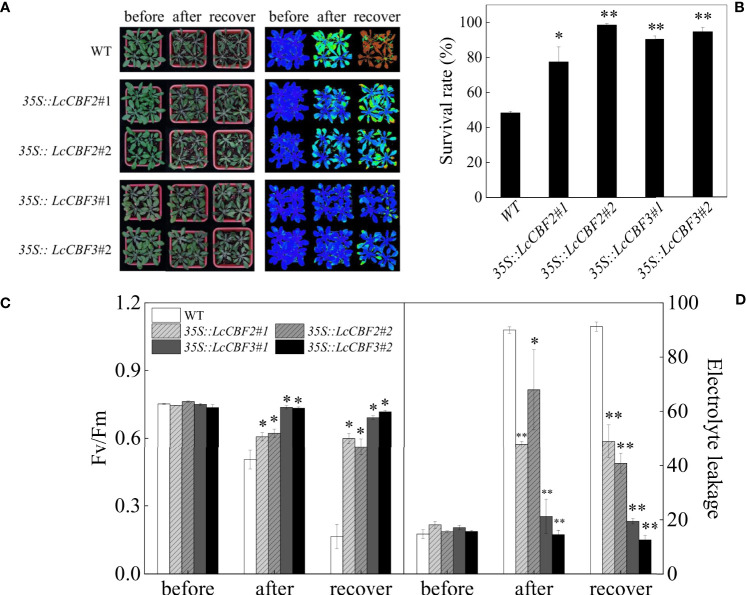
Overexpression of *LcCBF2* and *LcCBF3* improved the freezing tolerance of transgenic *Arabidopsis thaliana* plants. **(A)** response of wild-type (WT) and transgenic *Arabidopsis thaliana* over-expressing *LcCBF2* and *LcCBF3* to the freezing treatment; **(B)** Survival rates of plants after the freezing treatment; **(C)** comparisons of *Fv*/*Fm* and electrolyte leakage values **(D)** of freezing-treated WT and transgenic plants. The significant difference was determined by t test and indicated with * (at *p* < 0.05) and ** (at *p* < 0.01), respectively.

The *35S*::*LcCBF2/3* transgenic *Arabidopsis* plants were subjected to a freezing treatment to investigate their roles in modulating cold resistance. First, 2-week-old transgenic *Arabidopsis* seedlings grown on MS agar medium were subjected to the freezing treatment. After the freezing treatment, approximately half of wild-type plants died after a further 3 d under the non-stressed conditions (**Figure 5A**), whereas the *35S*::*LcCBF2* and *35S*::*LcCBF3* transgenic seedlings had a significantly higher survival rate at the same conditions (**Figure 5B**). Furthermore, a similar freezing treatment was applied to 4-week-old wild-type and transgenic plants, and chlorophyll fluorescence (indicates as *Fv*/*Fm* values to monitor PSII activity) and electrolyte leakage values of leaves were quantified to determine the freezing damage. The average *Fv*/*Fm* values detected with the chlorophyll fluorescence test were 0.75 for both the wild-type and *LcCBF* transgenic *Arabidopsis* lines at regular growing conditions (**Figure 5C**). After the freezing treatment, the *Fv*/*Fm* values remarkedly decreased in wild-type *Arabidopsis* leaves and showed the lowest value, followed by the values for *35S*::*LcCBF2* plants, whereas the *35S*::*LcCBF3* transgenic plants exhibited no significant differences in *Fv*/*Fm* values across the freezing treatment (**Figure 5C**). Accordingly, the average electrolyte leakage value of leaves from the *35S*::*LcCBF3* transgenic plants was the lowest after the treatment, followed by the values for *35S*::*LcCBF2* plants, whereas the wild-type plants showed a significantly higher values at the complete of freezing treatment (**Figure 5D**). It would be concluded that an increase of *LcCBF2* and/or *LcCBF3* expression could improve the freezing tolerance of plants.

**Figure 6:**
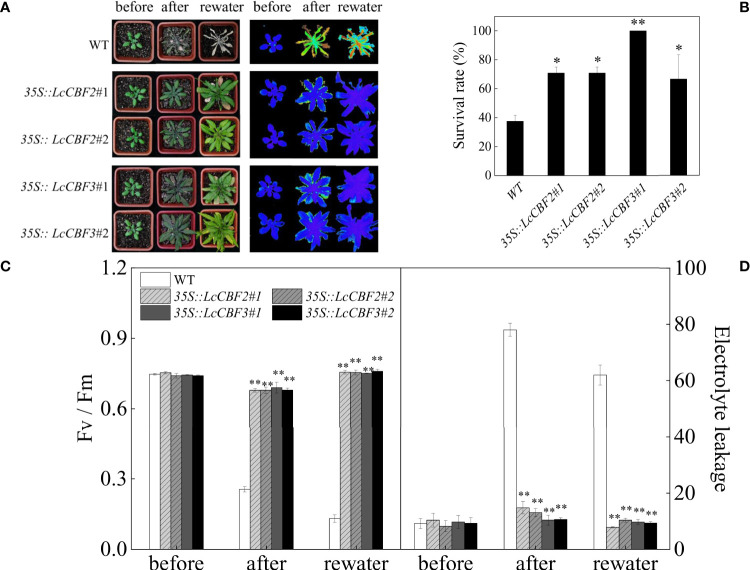
Overexpression of *LcCBF2* and *LcCBF3* improved the drought tolerance of transgenic *Arabidopsis thaliana* plants. **(A)** response of wild-type (WT) and transgenic *Arabidopsis thaliana* over-expressing *LcCBF2* and *LcCBF3* to the drought treatment; **(B)** Survival rates of plants after the drought treatment; **(C)** comparisons of *Fv*/*Fm* and electrolyte leakage values **(D)** of drought-treated WT and transgenic plants. The significant difference was determined by t test and indicated with * (at *p* < 0.05) and ** (at *p* < 0.01), respectively.

In addition, the effect of overexpression of *LcCBF2* and *3* on drought tolerance was tested. Individually potted wild-type and the selected *35S*::*LcCBF2* and *35S*::*LcCBF3* transgenic lines were subjected to a drought treatment by removal of watering for 16 d, and all lines were analyzed after a further 7 d of rewatering. More than 60% of wild-type plants were dead (**Figure 6A**), whereas the overexpression transgenic lines had a significantly higher survival rate at the completion of the treatment, indicating that both LcCBF2 and LcCBF3 could increase drought tolerance (**Figure 6B**). The reduction in *Fv*/*Fm* values was observed in both wild-type and transgenic plants attributed to the drought treatment, whereas the average *Fv*/*Fm* values remarkedly decreased in wild-type plants and could not be restored by rewatering (**Figure 6C**). Drought treatment also induced serious damage in membrane stability, resulting in a significant increase in electrolyte leakage in wild-type plants (**Figure 6D**). Taken together, the overexpression of *LcCBF2* or *LcCBF3* could improve the drought tolerance of transgenic *Arabidopsis thaliana*.

### Effects of *LcCBF2*, *LcCBF3* and *LcMFT* on flowering in *Arabidopsis*

**Figure 7:**
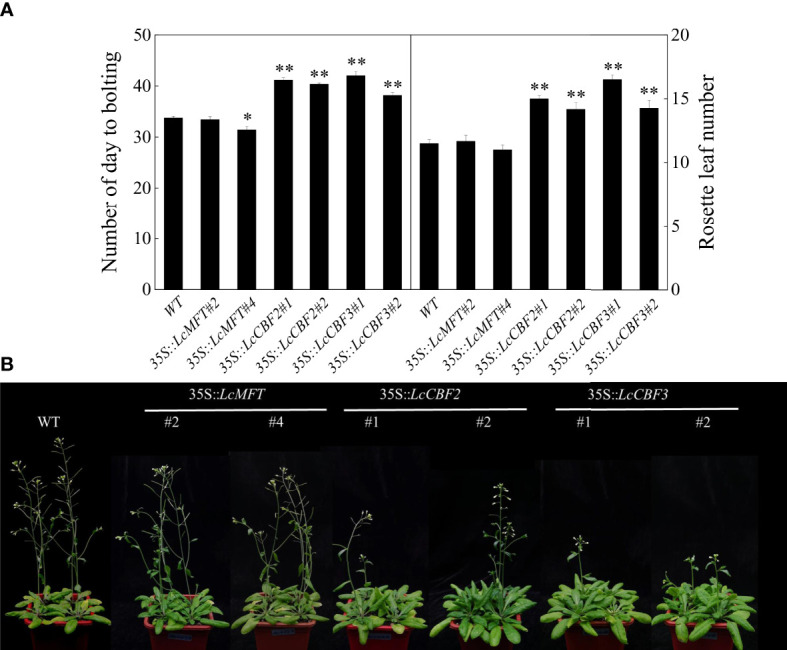
Effect of ectopic over-expressing *LcMFT* and *LcCBFs* on flowering. **(A)** Comparison of flowering time and number of rosette leaves at flowering between wild-type (WT) and *35S*::*LcMFT* and *35S*::*LcCBF*s transgenic lines; **(B)** Flowering phenotypes of WT and *35S*::*LcMFT* and *35S*::*LcCBFs* transgenic lines. The significant difference was determined by t test and indicated with * (at *p* < 0.05) and ** (at *p* < 0.01), respectively.

Moreover, the putative function of *LcCBF*s and *LcMFT* in the regulation of flowering time was analyzed. The time of bolting and the number of rosette leaves at that time were calculated as indicators of flowering time. Interestingly, overexpression of *LcMFT* could not influence the flowering time or alter the number of rosette leaves at flowering, although *LcMFT* is also a member of the PEBP family. By contrast, *35S*::*LcCBF2* and *35S*::*LcCBF3* transgenic lines took significant longer to bolt and had more cauline leaves at flowering under long-day conditions compared with the wild-type plants (**Figure 7A**). However, the morphological features of main inflorescence and rosette leaves were unaffected in those transgenic plants (**Figure 7B**). In conclusion, overexpression of *LcCBF2* and *LcCBF3*, instead of *LcMFT*, delayed the flowering time of *Arabidopsis thaliana*.

## Discussion

As a subtropical fruit tree, low ambient temperatures (< 18°C) or intermittent cold can activate the floral initiation in litchi (O’Hare, 2002). Consistently, in this study the potted litchi plants complete floral induction after 40-day exposure to 15°C in a green house. During low temperature-dependent litchi floral induction, the expression level of *LcCBF2* and *LcCBF3* decreased (**Figure 2**). It has been reported that the endogenous CBF protein levels are extremely low under warm growth conditions, but they dramatically accumulated in response to a 4°C treatment, reaching a peak at 6 h approximately (Lv et al., 2021). However, the accumulated effects of low ambient temperature result in a decreased expression of *CBF*s in *Arabidopsis*, due to the degradation of ICE1 protein (the upstream activator of *CBF*s) by the RING finger protein HIGH EXPRESSION OF OSMOTICALLY RESPONSIVE GENE1 (HOS1) (Dong et al., 2006; Lee et al., 2012). HOS1 can physically interact with ICE1 and causes the ubiquitination and subsequent degradation of ICE1 protein (Dong et al., 2006). HOS1 protein predominantly resides in the cytoplasm at normal growth temperatures, whereas HOS1 transfers into the nucleus and accumulated there in the presence of low temperature (Lee et al., 2001; Dong et al., 2006). Moreover, the recessive *hos1* mutation leads to enhanced induction of the *CBF* genes by low temperature and their downstream genes, while overexpression of *HOS1* represses the expression of *CBF*s as well as of their downstream cold-responsive genes, thereby supporting the reduction of *CBF* expression in litchi in response to floral inductive low temperature (Lee et al., 2001; Dong et al., 2006).

In addition, ectopic overexpression of *LcCBF2* or *LcCBF3* in *Arabidopsis* resulted in delayed flowering time and increased freezing and drought tolerance (**Figures 5**–**7**). In accordance with the notion, it has been demonstrated that overexpression of *CBF1*, *CBF2*, and *CBF3* in *Arabidopsis* causes delayed flowering and dwarf phenotypes, as well as alterations in indicators involved in increased freezing tolerance, such as accumulations of proline and sugar and transcriptional activation of *COR* genes (Gilmour et al., 2004). The delayed flowering may be partially attributed to the activation of *FLC* by the increased expression of *CBF*s, subsequently repressing the two flowering pathway integrators *FT* and *SOC1* (Seo et al., 2009). As the upstream regulator of CBFs, ICE1 also directly activates the gene encoding FLC, which represses *SOC1* expression. By contrast, SOC1 play as a transcriptional repressor of *ICE1* and *CBF* genes, inducing flowering with a reduction of freezing tolerance under floral promotive conditions (Seo et al., 2009; Lee et al., 2015).

In this report, we also showed the direct binding of LcCBF2/3 proteins to the *LcMFT in vivo* and *vitro* (**Figure 3**), as well as the involvement of the CBF-MFT module in fine-tuning of litchi flowering (**Figure 7**). The CBF/DREB1 transcription factors are able to specifically recognize a CRT/DRE (CCGAC) motif within the promoters of *COR* genes to mediate their expression. In *Arabidopsis*, according to the ChIP-seq analysis on a genome-wide scale, the binding region peaks of the three *CBF*s were all located 1,500-2,000 bp upstream of the transcription start site (TSS), with the highest frequency of peaks around the TSS itself. However, more than 50% of the binding sites for each CBF protein were located within the 1,000-bp promoter regions of the target genes (Song et al., 2021). Furthermore, RNA-seq data derived from individual *cbf1*, *cbf2* and *cbf3* mutants showed the over-lapping and distinct regulated genes by each CBF protein (Jia et al., 2016; Zhao et al., 2016; Shi et al., 2017; Song et al., 2021). In this study, one CRT/DRE cold response element was found in the proximal region of the promoter of *LcMFT*, and the protein-DNA interaction was exclusively observed between LcCBF2/3 and *LcMFT* promoter as indicated by the analysis of EMSA and Dual luciferase complementation assay (**Figure 3**).

MFT belongs to the PEBP family that includes *FT*-like subfamily, *TFL1*-like subfamily and *FT*-like subfamily. However, members within this family show distinct or opposite functions in regulating flowering time (Ahn et al., 2006). LcMFT, similar to the LcFT1 and LcTFL1, has been demonstrated to physically interact with the LcFD protein (Data submitted). The decrease of *LcCBF* and its target (*LcMFT*) during the litchi floral induction may inhibit the competitive interaction between LcMFT and LcFD, enhancing litchi flowering. Similar observations in other species also demonstrate the negative role of *MFT* homologs in controlling flowering time (Li et al., 2012; Bi et al., 2016). However, overexpression of *LcMFT* in *Arabidopsis* did not alter the flowering time significantly, indicating the potential weakened protein-protein interaction between LcMFT and AtFD, and/or the presence of other downstream player(s) of CBF protein involved in flowering (Yoo et al., 2004; Hou and Yang, 2016).

In conclusion, four *CRT/DRE BINDING FACTORS* (*CBF*) homologs were identified in litchi, of which *LcCBF1*, *LcCBF2* and *LcCBF3* decreased in response to floral inductive cold. A similar expression pattern was observed for the *MOTHER OF FT AND TFL1* homolog (*LcMFT*) in litchi. Furthermore, both LcCBF2 and LcCBF3 were found to bind to the promoter of the *LcMFT* gene to activate its expression. Ectopic overexpression of *LcCBF2* and *LcCBF3* in *Arabidopsis* caused delayed flowering and increased freezing and drought tolerance. Therefore, prolonged low temperatures facilitate litchi floral initiation partially by the suppression of *LcCBF2* and *LcCBF3.*

## Data availability statement

The datasets presented in this study can be found in online repositories. The names of the repository/repositories and accession number(s) can be found in the article/**Supplementary Material**.

## Author contributions

JS, HC, and XS contributed to the design of this work. Material preparation, data measurements and analyses were performed by XS, YY, SW, CW, and WS. The first draft of the manuscript was written by XS. The latest version of the manuscript was written by XS and JS. All authors contributed to the article and approved the submitted version.
