# Supplementary material for: Involvement of CBF in the fine-tuning of litchi flowering time and cold and drought stresses
Source: Front Plant Sci. 2023 Jun 12;14:1167458. doi: 10.3389/fpls.2023.1167458 (PMC10291182; doi:10.3389/fpls.2023.1167458)
Supplement: Supplementary file 1 [file Table_1.docx]

**Supplementary table 1 primers used in this study**

| Primers | Sequence of primers (5’-3’) |
| --- | --- |
| *LcMFT*pro-LUC-F | ATACGACTCACTATAGGGCGAATTGGGTACCATGATAAGAAACACTATTATG |
| *LcMFT*pro-LUC-R | CGGTGGCGGCCGCTCTAGAACTAGTGGATCCCCGAGCCATATCGATGGACGA |
| *LcCBF1*-SK-F | CGGTGGCGGCCGCTCTAGAACTAGTGGATCCATGGAGTTTTTGTCGAGCTATTCT |
| *LcCBF1*-SK-R | CGACGGTATCGATAAGCTTGATATCGAATTCTTATATTGAATAACTCCACAGTGA |
| *LcCBF2*-SK-F | CGGTGGCGGCCGCTCTAGAACTAGTGGATCCATGGTCATGTTCAGTCATCTATCA |
| *LcCBF2*-SK-R | CGACGGTATCGATAAGCTTGATATCGAATTCTCAAAATGAAAAACTCCACAATGAG |
| *LcCBF3*-SK-F | CGGTGGCGGCCGCTCTAGAACTAGTGGATCCATGGAACATAAAGATGAGTA |
| *LcCBF3*-SK-R | CGACGGTATCGATAAGCTTGATATCGAATTCTCAGTCACTCCACAAAGTCA |
| *LcCBF4*-SK-F | CGGTGGCGGCCGCTCTAGAACTAGTGGATCCATGAGTGAGGCACAAAACTC |
| *LcCBF4*-SK-R | CGACGGTATCGATAAGCTTGATATCGAATTCCTAACAATCCCAAAACAAAC |
| *LcCBF2*-pGEX4T1- F | GTTCCGCGTGGATCCCCGGAATTCATGGTCATGTTCAGTCAT |
| *LcCBF2*-pGEX4T1- R | TCAGTCACGATGCGGCCGCTCGAGTCAAAATGAAAAACTCCA |
| *LcCBF3*-pGEX4T1- F | GTTCCGCGTGGATCCCCGGAATTCATGGAACATAAAGATGAGT |
| *LcCBF3*-pGEX4T1- R | TCAGTCACGATGCGGCCGCTCGAGTCAGTCACTCCACAAAGT |
| *LcMFTpro* probe-F | GTGACATCAAGAGCCGACAAAGACGACCAC |
| *LcMFTpro* probe-R | GTGGTCGTCTTTGTCGGCTCTTGATGTCAC |
| *LcMFTpro* probe-mutant-F | GTGACATCAAGAGAAAAAAAAGACGACCAC |
| *LcMFTpro* probe-mutant-R | GTGGTCGTCTTTTTTTTCTCTTGATGTCAC |
| *LcMFT*pro-AbAi-F | GAAAAGCTTGAATTCGAGCTCATGATAAGAAACACTATTATG |
| *LcMFT*pro-AbAi-R | AGCACATGCCTCGAGGTCGACCCGAGCCATATCGATGGACGA |
| *LcMFT*-domain1pro*-*AbAi*-*F | GAAAAGCTTGAATTCGAGCTCAATTAAAAAATTGTATTCGTTT |
| *LcMFT*-domain1pro*-*AbAi*-*R | AGCACATGCCTCGAGGTCGACTTTTTTTTGGACAGCTTGTAA |
| *LcMFT*-domain2pro*-*AbAi*-*F | GAAAAGCTTGAATTCGAGCTCCCAATAAAAACAGGTTAATG |
| *LcMFT*-domain2pro*-*AbAi*-*R | AGCACATGCCTCGAGGTCGACGTCATTCGTGGTCGTCTTTG |
| *LcMFT*-domain3pro*-*AbAi*-*F | GAAAAGCTTGAATTCGAGCTCTCACCAGTAACATCAAGAGC |
| *LcMFT*-domain3pro*-*AbAi*-*R | AGCACATGCCTCGAGGTCGACATTACCTGAAAAATGATCGTG |
| *LcCBF2*-AD-F | GCCATGGAGGCCAGTGAATTCATGGTCATGTTCAGTCATCTA |
| *LcCBF2*-AD-R | CAGCTCGAGCTCGATGGATCCTCAAAATGAAAAACTCCACAA |
| *LcCBF3*-AD-F | GCCATGGAGGCCAGTGAATTCATGGAACATAAAGATGAGTA |
| *LcCBF3*-AD-R | CAGCTCGAGCTCGATGGATCCTCAGTCACTCCACAAAGTCA |
| *LcWRKY*-BD-F | ATGGCCATGGAGGCCGAATTCATGGGGACTGGGAAAGACGAG |
| *LcWRKY*-BD-R | ATGCGGCCGCTGCAGGTCGACTTACACTACGATTGGCTCTTT |
| *LcCBF2*-1302-F | GCTATGACCATGATTACGAATTCATGGTCATGTTCAGTCATCTATCAGAT |
| *LcCBF2*-1302-R | CAGGTCGACTCTAGAGGATCCTCAAAATGAAAAACTCCACAATGAG |
| *LcCBF3-*1302-F | GCTATGACCATGATTACGAATTCATGGAACATAAAGATGAGTACCAATCA |
| *LcCBF3*-1302-R | CAGGTCGACTCTAGAGGATCCTCAGTCACTCCACAAAGTCAAGTCTA |
| *LcMFT*-1302-F | GCTATGACCATGATTACGAATTCATGGCTCGGTCTCTAGACCCTCTGGTCG |
| *LcMFT*-1302-R | CAGGTCGACTCTAGAGGATCCTCAACGTTTTCTAACCGTTATTTCCCT |
| *LcFT1*-q-F | GCCGCGGTCTACTTTAATAGC |
| *LcFT1*-q-R | TCTTTGCTAGCTTTGACATGC |
| *LcMFT*-q-F | GTAATGGTGGATCCTGATGCT |
| *LcMFT*-q-R | TGTATGGCACCAACTCTTTCC |
| *LcActin*-q-F | ACCGTATGAGCAAGGAAATCACTG |
| *LcActin*-q-R | TCGTCGTACTCACCCTTTGAAATC |
| *At*actin-q-F | TCGGTGGTTCCATTCTTGCT |
| *At*actin-q-R | GCTTTTTAAGCCTTTGATCTTGAGAG |
| *LcFLC*-q-F | GCTGTGAACAGTGTTAATGCAG |
| *LcFLC*-q-R | TTCTAGCTCTGGTTTGTGTCAG |
| *LcSOC1-*q*-*F | *GATAGAACAACAGCTGGAGAGG* |
| *LcSOC1-*q*-*R | *GCTGCTCTTTCTCTTTCTCCTT* |
| *LcICE1-*q*-*F | AAAGAGGAGGATGAGTGTGAGTG |
| *LcICE1-*q*-*R | AGTAGCTCGATGGCGAATAGTAC |
| *LcHOS1-*q*-*F | CTGATAGAGATGATGGACCTTGG |
| *LcHOS1-*q*-*R | ATACCTATCCTCCACAGCAGATG |
| *LcCBF1-*q*-*F | *GAACAAGAAGTCCAGGATTTGG* |
| *LcCBF1-*q*-*R | *CTGTATGTCCTTCGCTTCGTTA* |
| *LcCBF2*-q-F | ACTCGCCACCCGATATACAG |
| *LcCBF2*-q-R | CTGCCATTTCTGGAGTAGGG |
| *LcCBF3-*q*-*F | TCTTCAACCTCAAGCGACCT |
| *LcCBF3-*q*-*R | GCGCGTCTCTTGAAACTTCT |
| *LcCBF4*-q-F | *ACCTCATCATCCTCATCTTCGT* |
| *LcCBF4*-q-R | *GCAGAGTCGTACTCGTAACTGG* |
