# Supplementary material for: Involvement of CBF in the fine-tuning of litchi flowering time and cold and drought stresses
Source: Front Plant Sci. 2023 Jun 12;14:1167458. doi: 10.3389/fpls.2023.1167458 (PMC10291182; doi:10.3389/fpls.2023.1167458)
Supplement: Supplementary file 2 [file Table_2.docx]

| **Gene Name** | **Species** | **Accession number** |
| --- | --- | --- |
| AtCBF1 | Arabidopsis (*Arabidopsis thaliana*) | AT4G25490 |
| AtCBF2 | Arabidopsis (*Arabidopsis thaliana*) | AT4G25470 |
| AtCBF3 | Arabidopsis (*Arabidopsis thaliana*) | AT4G25480 |
| AtCBF4 | Arabidopsis (*Arabidopsis thaliana*) | AT5G51990 |
| SlCBF1 | Tomato (*Solanum lycopersicum*) | Solyc03g026280 |
| SlCBF3 | Tomato (*Solanum lycopersicum*) | Solyc03g026270 |
| LcCBF1 | Litchi (*Litchi chinensis*) | LITCHI009273 |
| LcCBF2 | Litchi (*Litchi chinensis*) | LITCHI022951 |
| LcCBF3 | Litchi (*Litchi chinensis*) | LITCHI018796 |
| LcCBF4 | Litchi (*Litchi chinensis*) | LITCHI017181 |
|  |  |  |
| AtFT | Arabidopsis (*Arabidopsis thaliana*) | AT1G65480 |
| AtMFT | Arabidopsis (*Arabidopsis thaliana*) | AT1G18100 |
| AtTFL | Arabidopsis (*Arabidopsis thaliana*) | AT2G27550 |
| CsFT | Orange (*Citrus sinensis*) | Cs6g05140 |
| CsMFT | Orange (*Citrus sinensis*) | Cs2g06960 |
| CsTFL | Orange (*Citrus sinensis*) | Cs6g15000 |
| DlFT | Longan (*Dimocarpus longan*) | Dil.05g001470 |
| DlMFT | Longan (*Dimocarpus longan*) | Dil.15g013870 |
| DlTFL1 | Longan (*Dimocarpus longan*) | Dil.14g009880 |
| FaFT1 | Strawberry (*Fragaria ananassa*) | LC017713 |
| FaFT2 | Strawberry (*Fragaria ananassa*) | LC017714 |
| FaFT3 | Strawberry (*Fragaria ananassa*) | LC017715 |
| FaMFT | Strawberry (*Fragaria ananassa*) | KM435239 |
| FaTFL1 | Strawberry (*Fragaria ananassa*) | JN788264 |
| FvFT1 | Wild strawberry (*Fragaria vesca*) | NP_001266951 |
| FvFT2 | Wild strawberry (*Fragaria vesca*) | XM_004297225 |
| FvFT3 | Wild strawberry (*Fragaria vesca*) | XM_004295562 |
| GmBFTa | Soybean (*Glycine max*) | Glyma09g26550 |
| GmBFTb | Soybean (*Glycine max*) | Glyma16g32080 |
| GmFT1b | Soybean (*Glycine max*) | Glyma18g53690 |
| GmFT2b | Soybean (*Glycine max*) | Glyma16g26660 |
| GmFT3a | Soybean (*Glycine max*) | Glyma16g04840 |
| GmFT3b | Soybean (*Glycine max*) | Glyma16g26690 |
| GmFT4 | Soybean (*Glycine max*) | Glyma08g47810 |
| GmFT5a | Soybean (*Glycine max*) | Glyma16g04830 |
| GmFT5b | Soybean (*Glycine max*) | Glyma19g28400 |
| GmMFT | Soybean (*Glycine max*) | Glyma05g34030 |
| GmTFL1-2a | Soybean (*Glycine max*) | Glyma10g08340 |
| GmTFL1-2b | Soybean (*Glycine max*) | Glyma13g22030 |
| GmTFL1a | Soybean (*Glycine max*) | Glyma03g35250 |
| GmTFL1b | Soybean (*Glycine max*) | Glyma19g37890 |
| HvMFT | Barley (*Hordeum vulgare* ) | BAH24198 |
| HvTFL1 | Barley (*Hordeum vulgare* ) | BAH24198 |
| JcMFT1 | Barbadosnut (*Jatropha curcas*) | KC874668 |
| JcMFT2 | Barbadosnut (*Jatropha curcas*) | KF944352 |
| LcFT1 | Litchi (*Litchi chinensis*) | LITCHI002331 |
| LcFT2 | Litchi (*Litchi chinensis*) | LITCHI006928 |
| LcMFT | Litchi (*Litchi chinensis*) | LITCHI029325 |
| LcTFL1 | Litchi (*Litchi chinensis*) | LITCHI018838 |
| MdTFL1 | Apple (*Malus domestica* ) | MD12G1023900 |
| MtMFT | Apple (*Malus domestica* ) | Medtr8g106840 |
| OsFT | Rice (*Oryza sativa*) | Os06g06320 |
| OsMFT1 | Rice (*Oryza sativa*) | Os06g30370 |
| OsMFT2 | Rice (*Oryza sativa*) | Os01g02120 |
| PtMFT | Black cottonwood (*Populus trichocarpa*) | DQ310725 |
| TaMFT | Bread wheat (*Triticum aestivum* ) | BAK78908 |
| VvFT | Grape (*Vitis vinifera*) | DQ871590 |
| VvMFT | Grape (*Vitis vinifera*) | DQ871594 |
| VvTFL1A | Grape (*Vitis vinifera*) | DQ871591 |
| VvTFL1B | Grape (*Vitis vinifera*) | DQ871592 |
| ZCN9 | Maize (*Zea mays*) | ABX11011 |
| ZCN10 | Maize (*Zea mays*) | ABW96233 |
| ZCN11 | Maize (*Zea mays*) | ABX11013 |
